# Medical and Physical *Intelligence*

**Published:** 1800-01

**Authors:** 


### MEDICAL AND PHYSICAL

### *INTELLIGENCE,*

### ( Original and Selected. )

CITIZEN VAUQUELIN communicated to the Philomatic Society an obfervation on cryftals, formed by a mixture of oil of rofemary with a folution of gold. He obferved at the bottom of the veffel feveral clufters of tranfparent needles, confifting of quadrangular prifms terminated by pyramids with four fides. This fubftance was brittle, and had the tafte of oil of rofemary: the different experiments to which he fubmitted it were not fufficient to difcover its nature; he only afcertained that it was not camphire, though M. Prouft afferts, that he has found that fubftance in feveral volatile oils.—*Rapport Général des Travaux de la Société Philomatique de Paris*, v. i. p. 47.

Cit. VAN MONS tranfmitted to the Society the refult of an experiment on melting fome cauftic potafs with metallic oxyds, and particularly the oxyd of mercury, in which thefe fubftances emitted a nitrous odour, whence he concluded that the potafs had azote for its conftituent principle. Citizens Vauquelin and Hecht, having been requefted by the Society to verify this affertion, declared that they difcovered no fimilar phænomenon. They remarked that the oxyd of mercury was reduced, without difcharging a fingle bubble of oxygen gas, when they ufed a retort of flint glafs, but the liquor then became green; when expofed to the air, it changed to a violet colour, and depofited a brown fediment, which they difcovered to be manganefe. The potafs contained a quantity of filex; hence they concluded that the oxygen of the mercury had paffed into the manganefe; fo that the latter had been diffolved by the potafs which corroded the retort. To confirm this explanation, they repeated the experiment in a porcelaine retort, when the oxygen gas was difcharged, but the fubftance did not change its colour. In both experiments the potafs underwent no change in its nature, and they obferved, that even if the nitric acid fhould refult from the procefs, it would neceffarily remain combined with the potafs, unlefs it was reduced to its elements, oxygen and azotic gas, in a very high temperature.—*Ibid.* p. 49.

Cit. Vauquelin and Brongniart were appointed by the Society, to authenticate the affertion of Valli, that the gluten of wheat and the animal fibre, when treated with the acetic acid, change their nature; nature; the wheat turning to feculence, and the animal fibre to jelly; and that flour is one of the alimentary fubftances which contains the greateft proportion of phofphat of lime. They found, on the contrary, that gluten and animal fibre, when triturated with acetic acid, readily diffolved, and after having continued fome time in that ftate, a few drops of alkali made them re-appear with all their properties. They alfo difcovered, that a pound of flour contains only 84 grains of calcareous phofphat.—*Ibid.* p. 51.

---

Cit. Brongniart has repeated the experiments made by Cit. Benedict Provoft, of Montauban, on the means of rendering the effluvia of odoriferous bodies perceptible to the eye. Thefe experiments tend to prove, that all odoriferous bodies are furrounded with an atmofphere of an elaftic fluid which is forcibly difengaged. —*Ibid.* p. 52.

---

Cit. Bouillon Lagrange communicated a memoir on *camphire* and *camphoric acid.* He has difcovered that camphire is a volatile oil, rendered concrete by carbon; that, on being diftilled with alum in a retort, the carbon and volatile oil may be obtained feparately; and that camphire, when treated with nitric acid, produced an acid different from that of all known vegetables.—*Ibid.*

---

The fame Member informed the Society of a memoir read by him to the National Inftitute, on the analyfis of cork, and on the nature of the peculiar acid which he obtained from it, by means of the nitric acid. He alfo extracted from this fubftance a refinous matter, which by its foftnefs had great analogy to vegetable wax.—*Ibid.*

---

Cit. Lamark read to the Society feveral memoirs on the effential particles of compound bodies, the immutability of their forms, and the unity or identity of their nature. He eftablifhed as a principle, that all homogeneous natural bodies were compofed of particles exactly fimilar to each other, which remain alike as long as the number, the proportions, and the arrangement of the principles which compofe them remain the fame; that the aggregate of thefe particles conftitutes the perceptible maffes of bodies; that in the act of their combination, a difunion of the principles of the integral particles takes place; that the combination being once made, the component parts again form homogeneous bodies, the integral particles of which have particular and conftant forms, as well as thofe which compofe them; and that mere aggregation occafions the heterogeneity of bodies. From thefe affertions he concludes, that twofold and threefold component parts do not exift in the manner which modern chemifts have maintained, and that the refiduum or produce of chemical operations is not contained in bodies fubmitted to analyfis.—*Ibid.* p. 56.

Cit. Lacroix read the refult of fome experiments which he had begun on the analyfis of fnow water: he obferves, that the elaftic fluid which efcapes on melting fnow, contains only twenty-three parts of oxygen gas.—*Ibid.* p. 57.

Cit. Lelievre communicated a report made to the Committee of Public Welfare, on the extraction of foda from marine falt. He called the attention of the Society to the procefs ufed at Javelle, to extract foda from the fulphat of foda, by means of iron reduced to fmall pieces. On the procefs employed by Citizens Leblanc and Dize, in which the fulphat of foda is decompofed by means of carbon and chalk, and on that invented by Citizens Malherbe and Atenas, which confifts of immediately decompofing the muriat of foda by the fulphat of iron, he obferved, that the laft method appears the moft advantageous, on account of the great abundance of pyrites which may be employed for this purpofe.—*Ibid.*

A memoir having been prefented to the Society, in which feveral means were propofed for the prefervation of water in long voyages, and particularly that of violently fhaking ftagnated water to combine with it a large quantity of atmofpheric air, Cit. Vauquelin announced, that an individual had fuccefsfully ufed lime water, with which he wafhed the infide of the cafks, and which, by changing into calcareous carbonat, effectually prevented putrefaction.—*Ibid.* p. 61.

Cit. Lefêbure ftated, that having bought fpoons which he fuppofed to be tin, his family, foon after ufing them, were afflicted with colics; in confequence of which he refolved to analyze their compofition. He afcertained by his experiments, that the fpoons were made of feventy parts of tin, twenty of lead, and ten of copper; hence he attributed their pernicious effects entirely to the lead, and added fome obfervations on the danger of making culinary utenfils of thofe combined metals.—*Ibid.* p. 63.

Cit. Girod Chantran communicated an experiment, which tended to prove the antifeptic property of beer. He put a piece of meat, which was entirely fly-blown, into a veffel that contained a certain quantity of beer; the liquor became tainted with a putrid fmell; and after it was drawn off, the meat was fufficiently purified to make good broth, and had not a difagreeable tafte. Several experiments made by the fame phyfician, convinced him of the antifeptic virtues of that beverage.—*Ibid.* p. 68.

Cit. Bouvier read a memoir on the procefs employed to extract oil of juniper from the *juniperus oxiacantha*, and on the difference between that oil and the oil of gabian, with which it is confounded in commerce. This oil is ufed for the cure of cutaneous difeafes of animals.—*Ibid.*

Cit.

Cit. Hauy ftated the means which he had fuccefsfully employed, to preferve the natural colour in the petals of a great number of dryed flowers. It was only neceffary to immerfe them for fome minutes in alcohol. The colours at firft faded, but in a fhort time they refumed their natural tint, which remained permanently fixed. The author is fully convinced of the fuccefs of this experiment, as he made it ten years ago on the flowers of various plants, particicularly the *Viola odorata, Geranium fanguineum,* and *Vicia dumetorum. Ibid.* p. 69.

---

M. Lucius, an apothecary at Cleves, having filled a jar with finely pulverized hæmatites, obferved that the particles which adhered to the furface of the glafs, had affumed a black colour and metallic luftre. There remains no doubt, that this phænomenon muft be afcribed to the influence of light, as only the minute particles of the hæmatites, which had been expofed to the rays of light through the glafs, underwent this change; but thofe in the middle of the veffel remained in the fame ftate as before. The opinion of M. Lucius is therefore very plaufible, that an imperfect reduction of the metal may have taken place, fimilar to what happens with the luna cornea. *Göttling's Tafchenb.* 1797, p. 110.

---

When Dr. Scherer was preparing oxydated azote for chemical experiments, by diffolving tinfoil and zinc in a much diluted nitrous acid, he remarked the fingular phænomenon, that though this gas was neither affected by oxygen nor nitrous gas, yet ignited phofphorus, as well as a lighted match, continued to burn in it with rapidity. *Gren's Journal,* B. III. p. 312.

From a number of experiments, inftituted to difcover the affinity between oxygenated muriatic acid and earths, Prof. Trommsdorf was convinced, that this acid entered into no combination with magnefia; but with lime, it formed a falt which confifted of triangular, acuminated, and radiated diverging prifms. This falt, during its folution in water, produced an increafed temperature, and was different in its other properties from the muriat of lime. Argilla yielded a falt that could not be cryftallized, and was likewife different from the muriat of that earth. *Journ. der Pharm.* B. III. p. 105.

---

The fame chemift repeated the experiments of M. Osburg, to difcover whether lime were a conftituent of the fixed vegetable alkali, and whether magnefia effentially contributed to the formation of mineral alkali. When thefe falts were expofed to a red heat in earthen and porcelain crucibles, and afterwards diffolved in diftilled water, fome filex and argilla were feparated. But on expofing the cryftallized carbonat of vegetable alkali, and likewife fome cryftals of foda in powder, to a red heat, twelve different times in a filver crucible, and diffolving them as often in diftilled water, the Prof. could not difcover the fmalleft particle of earth. He is confequently induced to believe, that we are not yet enabled to decompose the fixed alkalies into their constituent parts. *Journ. der Pharm.* B. III. p. 173.

