# Erratum

**Published:** 1800-02

**Authors:** 


### ERRATUM.

No. XI. page 92, line 6, for *cows*, read *cases*.

